# Supplementary material for: Defence versus growth in a hostile world: lessons from phage and bacteria
Source: R Soc Open Sci. 2020 Sep 16;7(9):201118. doi: 10.1098/rsos.201118 (PMC7540767; doi:10.1098/rsos.201118)
Supplement: Supplementary material [file rsos201118supp1.pdf]

# Supplementary Material for: Defence versus growth in a hostile world - Lessons from phage and bacteria

Rasmus Skytte Eriksen[1] & Sandeep Krishna[2]

[1] Niels Bohr Institute, University of Copenhagen, Copenhagen, Denmark

[2] Simons Centre for the Study of Living Machines, National Centre for Biological Sciences, Tata Institute of Fundamental Research, Bangalore, India.

## S1 Parameter values used in simulations

Throughout the paper, we use the parameter values listed in the table below unless otherwise specified.

| **Name** | **Value** | **Description** |
|---|---|---|
| C | $10^8$ | Carrying capacity of the ecosystem |
| $\eta$ | $10^{-8}$ | Adsorption rate of the phages |
| $\alpha$ | 0.2 | Death rate of the bacteria |
| $\beta$ | 50 | Burst size of the phages |
| $\delta$ | 0.2 | Decay rate of the phages |
| $T$ | $10^4$ | Interval between bacterial additions |
| $lb$ | 4 | log 10 of lower bound for omega |
| $\mu$ | 4 | Parameter controlling correlation |
| $t$ | $2 - \left(\frac{1}{2}\right)^{\left(\frac{1}{\mu}-1\right)}$ | Scaling factor for the growth rates |
| $M$ | 5 | Number of starting species |

Table S1: Default parameter values used in the simulations.

## S2 Generating correlated RM systems

When generating a correlated $(\gamma, \omega)$ pair in our simulations we use the following algorithm:

1. Draw $n$ from a Poisson distribution with mean $\mu$.
2. For each $r = 1, 2, \ldots, n$, draw two independent and identically distributed numbers $u_{1r}$ and $u_{2r}$ from a uniform distribution between $0 \leq u \leq 1$.
3. Compute $\gamma_r$ and $\omega_r$ defined as:
   $\omega_r = 10^{-\frac{lb}{\mu} u_{1r}}$
   $\gamma_r = 1 - t \cdot u_{2r}$
4. Compute the $(\gamma, \omega)$ pair by:
   $\gamma = \prod_{r=1}^{n} \gamma_r$
   $\omega = \prod_{r=1}^{n} \omega_r$

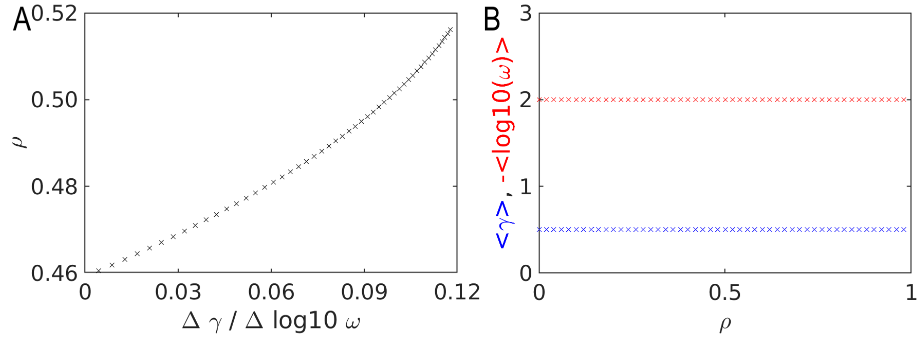


Figure S1: Change of correlation coefficient and marginal expectation values. In the main text figure 3, we investigate how the underlying distribution $\Pr(\gamma_i, \omega_i)$ changes the dynamics. Here we show (A) how the correlation coefficient changes as we change the marginal distributions in figure 3A, and (B) how the marginal distribution change as we change the correlation coefficient in figure 3B.

Here $lb$ sets the lower bound on $\omega$, and $\mu$ sets the mean of $\gamma$ as well as the correlation coefficient (see table S1 for default values).

This somewhat indirect method to generate correlations was used for historical reasons - arising from a separate study of strains containing multiple independent RM systems. In this context, we emphasise that this should not be taken as a proposed mechanism for why such correlations arise in bacteria with RM systems; it is simply one parametrization of a $\Pr(\gamma, \omega)$ distribution that exhibits correlations and conveniently allows us to change the "steepness" of the cost of RM systems by varying the parameter $t$.

In the main text, we compare such correlated $(\gamma, \omega)$ pairs with uncorrelated pairs and, to allow direct comparison, the expectation values should be identical for the uncorrelated pairs and the correlated pairs. We achieve this by setting $t = 2 - \left(\frac{1}{2}\right)^{\left(\frac{1}{\mu}-1\right)}$ which is derived by setting $\langle(1-tu)^{\mu}\rangle = \langle u \rangle$ when $\langle u \rangle = \frac{1}{2}$

## S2.1 Changing the cost of RM systems

In the main text, we test the effect of changing the "steepness" of the distribution by scaling the growth rates. This is done by sweeping the parameterization variable $t$. This parameter changes the marginal expectation values while keeping the correlation coefficient $\rho$ almost unchanging. Across the sweep of parameter $t$, the "slope" of the distribution changes – we quantify the slope as $\Delta\gamma/\Delta \log 10(\omega)$ – and the correlations drop from $\rho \approx 0.51$ at the shallowest distribution to $\rho \approx 0.46$ at the steepest distribution (see figure S1 for details).

## S2.2 Changing the correlation coefficient

In our investigation, we also test the effect of changing the correlation coefficient without changing the marginal expectation values. Here we use a multivariate normal distribution to draw the values of $\gamma$ and $\log 10(\omega)$.

The multivariate normal distribution $\Pr_{MVN}(\gamma, log10(\omega))$ is defined by a location vector $\vec{\mu}$, which sets the means of the marginal distributions:

$$\vec{\mu} = \begin{bmatrix} 0.5 \\ -2 \end{bmatrix} \tag{S1}$$

By defining $\vec{\mu}$ so, the drawn growth rates will have mean 0.5 and the drawn $\omega$ values will have mean $10^{-2}$. The distribution is further defined by a covariance matrix $\vec{\Sigma}$ which defines the spread of each of the marginal distribution and the correlations between the drawn values:

$$\vec{\Sigma} = \begin{bmatrix} \sigma_{\gamma}^2 & \rho\sigma_{\gamma}\sigma_{\log 10(\omega)} \\ \rho\sigma_{\gamma}\sigma_{\log 10(\omega)} & \sigma_{\log 10(\omega)}^2 \end{bmatrix} \tag{S2}$$

Where $\sigma_\gamma = 0.125$ and $\sigma_{\log 10(\omega)} = 0.75$, defines the marginal spreads, and $\rho$ is the Pearson correlation coefficient. Note that we truncate the distribution by redrawing pairs where either $\gamma < 0$ or $\log 10(\omega) > 0$.

We show in figure S1B that, as expected, the means of the marginal distributions do not change as we sweep the correlations coefficient from 0 to 1.

# S3 Analytical solution to the model

To begin, we remind the reader that the model equations we want to find the steady-state of are the following:

$$\dot{b_i} = \gamma_i b_i(1 - B/C) - \eta_i b_i p_i - \eta_i \omega_i b_i(P - p_i) - \alpha b_i \tag{S3}$$
$$\dot{p_i} = \eta_i \beta_i b_i p_i + \eta_i \omega_i \beta_i b_i(P - p_i) - \eta_i p_i B - \delta_i p_i \tag{S4}$$

In the steady state, the growth and decay terms balance:

$$b_i\left(\gamma_i(1 - B/C) - \alpha\right) = \eta_i b_i p_i + \eta_i \omega_i b_i(P - p_i) \tag{S5}$$
$$\eta_i \beta_i b_i p_i + \eta_i \omega_i \beta_i b_i(P - p_i) = \eta_i p_i B + \delta_i p_i \tag{S6}$$

We start by combining eq. (S3) and eq. (S4)

$$\beta_i b_i\left(\gamma_i(1 - B/C) - \alpha\right) = \eta_i p_i B + \delta_i p_i \tag{S7}$$

We then rearrange eq. (S5) and assume $p_i > 0$ to obtain the ratio:

$$\frac{b_i}{p_i} = \frac{\eta_i B + \delta_i}{\beta_i\left(\gamma_i(1 - B/C) - \alpha\right)} \tag{S8}$$

Next, we isolate $p_i$ in (S3) when $b_i > 0$:

$$\gamma_i(1 - B/C) - \alpha = \eta_i p_i + \eta_i \omega_i P - \eta_i \omega_i p_i \tag{S9}$$
$$\gamma_i(1 - B/C) - \alpha = \eta_i(1 - \omega_i)p_i + \eta_i \omega_i P \tag{S10}$$
$$p_i = \frac{\gamma_i(1 - B/C) - \alpha - \eta_i \omega_i P}{\eta_i(1 - \omega_i)} \tag{S11}$$
$$p_i = \frac{\gamma_i(1 - B/C)}{\eta_i(1 - \omega_i)} - \frac{\alpha}{\eta_i(1 - \omega_i)} - P\frac{\omega_i}{(1 - \omega_i)} \tag{S12}$$

The total phage mass is then found by perform a sum on eq. (S10):

$$P = (1 - B/C)\sum_j \frac{\gamma_j}{\eta_j(1 - \omega_j)} - \alpha \sum_j \frac{1}{\eta_j(1 - \omega_j)} - P\sum_j \frac{\omega_j}{(1 - \omega_j)} \tag{S13}$$
$$P = (1 - B/C)\frac{\sum_j \frac{\gamma_j}{\eta_j(1-\omega_j)}}{1 + \sum_j \frac{\omega_j}{(1-\omega_j)}} - \alpha\frac{\sum_j \frac{1}{\eta_j(1-\omega_j)}}{1 + \sum_j \frac{\omega_j}{(1-\omega_j)}} \tag{S14}$$

Next we isolate $b_i$ in eq. (S4):

$$\eta_i \beta_i b_i p_i + \eta_i \omega_i \beta_i b_i(P - p_i) = \eta_i p_i B + \delta_i p_i \tag{S15}$$
$$b_i p_i(1 - \omega_i) + b_i \omega_i P = p_i B\frac{1}{\beta_i} + p_i\frac{\delta_i}{\eta_i \beta_i} \tag{S16}$$

We assumed $p_i > 0$, so we can divide through by $p_i$.

$$b_i(1-\omega_i) + \frac{b_i}{p_i}\omega_i P = B\frac{1}{\beta_i} + \frac{\delta_i}{\eta_i\beta_i} \tag{S17}$$

We then insert eq. (S6) into eq. (S15).

$$b_i(1-\omega_i) + \frac{\eta_i B + \delta_i}{\beta_i\left(\gamma_i(1-B/C)-\alpha\right)}\omega_i P = B\frac{1}{\beta_i} + \frac{\delta_i}{\eta_i\beta_i} \tag{S18}$$

We can now isolate $b_i$ in eq. (S16):

$$b_i = B\frac{1}{\beta_i(1-\omega_i)} + \frac{\delta_i}{\eta_i\beta_i(1-\omega_i)} - \frac{\eta_i B + \delta_i}{\beta_i\left(\gamma_i(1-B)-\alpha\right)}\frac{\omega_i}{1-\omega_i}P \tag{S19}$$

The total number of bacteria is then found by summing over the $b_i$'s.

$$B = B\sum_j \frac{1}{\beta_j(1-\omega_j)} + \sum_j \frac{\delta_j}{\eta_j\beta_j(1-\omega_j)} - \sum_j \frac{\eta_j B + \delta_j}{\beta_j\left(\gamma_j(1-B/C)-\alpha\right)}\frac{\omega_j}{1-\omega_j}P \tag{S20}$$

By inserting our expression for $P$ (eq. (S12)) we obtain the self consistent equation for $B$.

$$\begin{aligned} B =& B\sum_j \frac{1}{\beta_j(1-\omega_j)} + \sum_j \frac{\delta_j}{\eta_j\beta_j(1-\omega_j)} \\ &- \sum_j \frac{\eta_j B + \delta_j}{\beta_j\left(\gamma_j(1-B/C)-\alpha\right)}\frac{\omega_j}{1-\omega_j}\Bigg((1-B/C)\frac{\sum_j \frac{\gamma_j}{\eta_j(1-\omega_j)}}{1+\sum_j \frac{\omega_j}{(1-\omega_j)}} \\ &- \alpha\frac{\sum_j \frac{1}{\eta_j(1-\omega_j)}}{1+\sum_j \frac{\omega_j}{(1-\omega_j)}}\Bigg) \end{aligned} \tag{S21}$$

Finally, we can rearrange eq. (S16) to simple expression for the $b_i$'s.

$$b_i = \frac{B + \delta_i/\eta_i}{\beta_i(1-\omega_i)}\left(1 - \frac{\eta_i\omega_i P}{\left(\gamma_i(1-B/C)-\alpha\right)}\right) \tag{S22}$$

# S4 Performance of the iterative algorithm

The above analytical solution allows us to use a simplified, iterative algorithm to predict the results from the full model.

## S4.1 Comparing with the simulated evolution

In figure S2, we run the iterative algorithm in conditions which mimic those use full dynamical model shown in figure 2. The overall behaviour is remarkably similar to that found in figure 2.

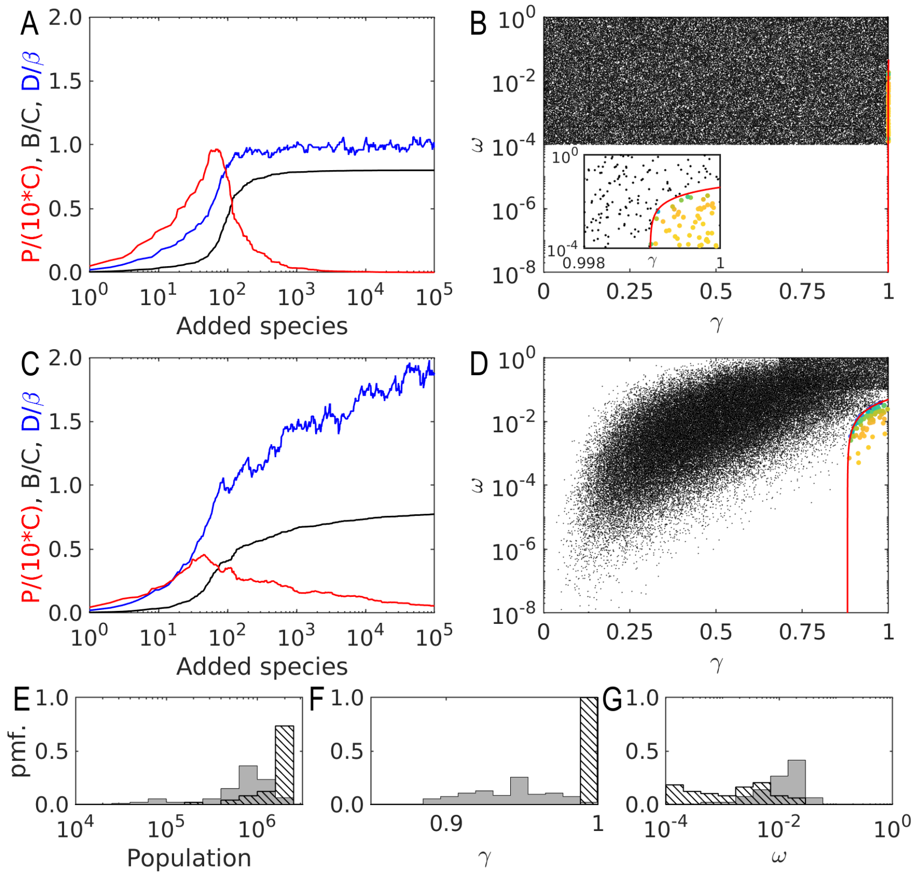


Figure S2: The iterative algorithm on an open system. We use our iterative algorithm to model the simulated evolution of figure 2B. We test the algorithm using an uncorrelated distribution for the RM systems (A-B) and correlated RM systems (C-D). Both cases are qualitatively similar to the results from the full dynamical model. (E-F) shows the distributions of (E) $\gamma$ and (F) $\omega$ at the end of the simulation with (un)correlated RM systems shown as (hatched) solid bars.

## S4.2 Finite species

We also more directly compare the predictions of the dynamical system with the predictions of our iterative map. To do so, we limit the number of possible $(\gamma, \omega)$ pairs available in the system. In doing so, the dynamical system will revisit $(\gamma, \omega)$ pairs until a steady-state is reached, rather than continuously generating new $(\gamma, \omega)$ pairs which prevent the system from ever settling down fully. Since the dynamical system now visits a known set of $(\gamma, \omega)$ pairs, we can directly compare the performance of our map with the dynamical system for different set sizes (see figure S3). Using the setup described above, we vary the number of $(\gamma, \omega)$ pairs from 10 to 1000 and compare the prediction between the two methods in two conditions: (i) using the same $(\gamma, \omega)$ values in both methods and (ii) using different realizations of $(\gamma, \omega)$ values from the same distribution. In the former case, we see an almost perfect match between the two methods while we in the latter case

see good agreement for the diversity and total bacterial population but some variation in the total phage population.

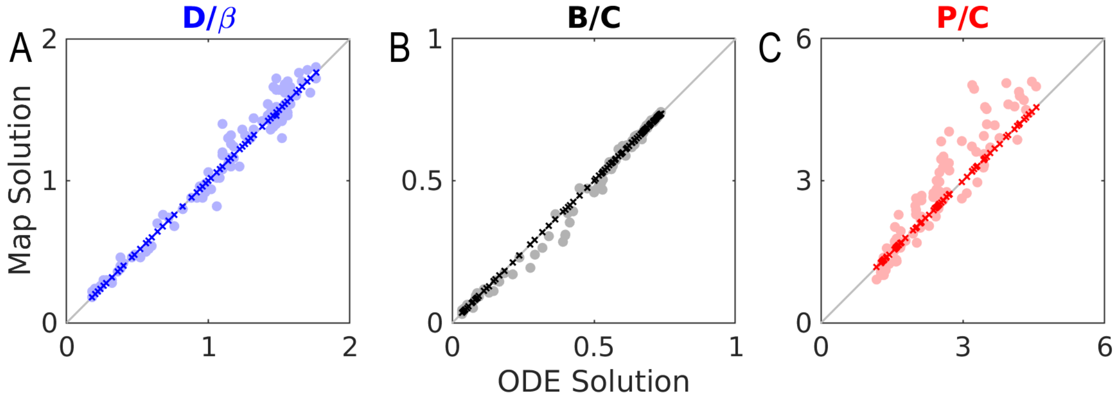


Figure S3: Comparison of the map and the dynamical system using same $(\gamma, \omega)$ values (dark crosses) and identically distributed $(\gamma, \omega)$ values (light circles). (A) $D/\beta$, (B) $B/C$, (C) $P/C$.

## S5 High diversity with the uncorrelated RM systems

From our steady-state solution in section S3, we now understand that a high diversity state can be induced by limiting the number of high $\gamma$ - low $\omega$ species. This means that we should be able to get a high diversity state from an uncorrelated distribution if the imperfections are drawn from the more limited interval: $10^{-2}$ to 1. In figure S4, we test this scenario and find that in fact we do get diversity substantially higher than $\beta$ under these conditions.

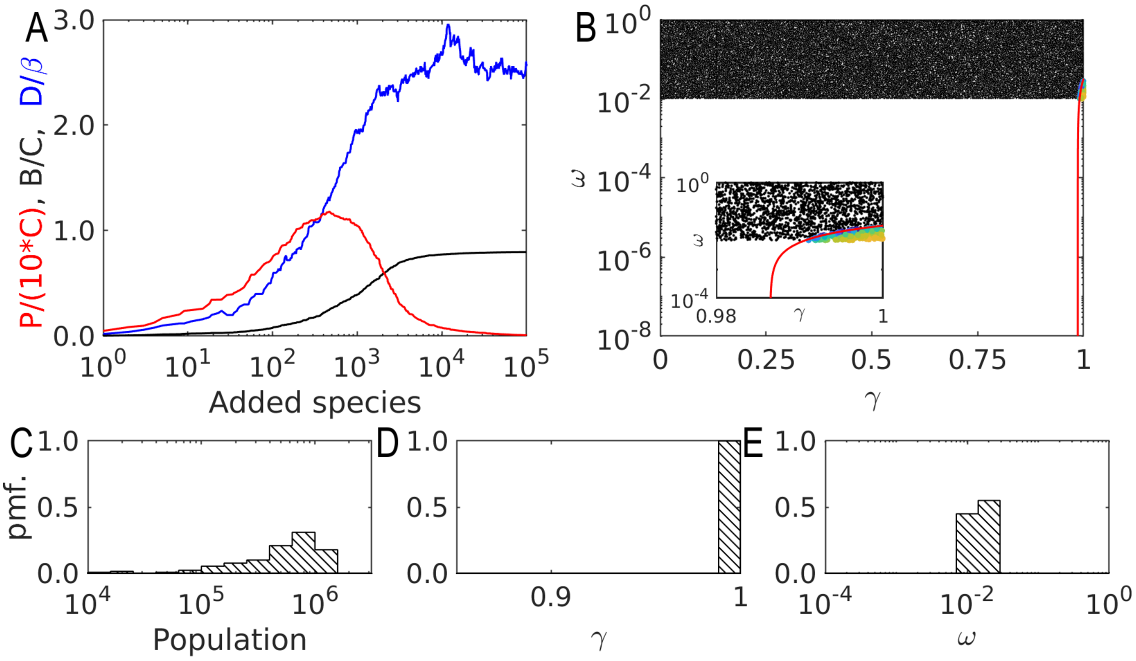


Figure S4: High diversity with uncorrelated RM systems. Using the iterative algorithm, we use an uncorrelated $\gamma - \omega$ distribution where the RM imperfections $\omega$ have values between $10^{-2}$ and 1. (A) Simulated evolution. (B) $\gamma - \omega$ distribution (C-E) Final distributions.
